# Making the most of all data: Combining non‐genotyped and genotyped potato individuals with HBLUP

**DOI:** 10.1002/tpg2.20056

**Published:** 2020-09-29

**Authors:** Salej Sood, Zibei Lin, Brittney Caruana, Anthony T. Slater, Hans D. Daetwyler

**Affiliations:** ^1^ Agriculture Victoria, AgriBio Centre for AgriBioscience 5 Ring Road Bundoora VIC 3083 Australia; ^2^ Division of Crop Improvement ICAR‐Central Potato Research Institute Shimla Himachal Pradesh 171001 India; ^3^ School of Applied Systems Biology La Trobe University Bundoora VIC 3083 Australia

## Abstract

Using genomic information to predict phenotypes can improve the accuracy of estimated breeding values and can potentially increase genetic gain over conventional breeding. In this study, we investigated the prediction accuracies achieved by best linear unbiased prediction (BLUP) for nine potato phenotypic traits using three types of relationship matrices pedigree ABLUP, genomic GBLUP, and a hybrid matrix (**H**) combining pedigree and genomic information (HBLUP). Deep pedigree information was available for >3000 different potato breeding clones evaluated over four years. Genomic relationships were estimated from >180,000 informative SNPs generated using a genotyping‐by‐sequencing transcriptome (GBS‐t) protocol for 168 cultivars, many of which were parents of clones. Two validation scenarios were implemented, namely “Genotyped Cultivars Validation” (a subset of genotyped lines as validation set) and “Non‐genotyped 2009 Progenies Validation”. Most of the traits showed moderate to high narrow sense heritabilities (range 0.22–0.72). In the Genotyped Cultivars Validation, HBLUP outperformed ABLUP on prediction accuracies for all traits except early blight, and outperformed GBLUP for most of the traits except tuber shape, tuber eye depth and boil after‐cooking darkening. This is evidence that the in‐depth relationship within the **H** matrix could potentially result in better prediction accuracy in comparison to using **A** or **G** matrix individually. The prediction accuracies of the Non‐genotyped 2009 Progenies Validation were comparable between ABLUP and HBLUP, varying from 0.17–0.70 and 0.18–0.69, respectively. Better prediction accuracy and less bias in prediction using HBLUP is of practical utility to breeders as all breeding material is ranked on the same scale leading to improved selection decisions. In addition, our approach provides an economical alternative to utilize historic breeding data with current genotyped individuals in implementing genomic selection.

**Abbreviations**
ABLUP: pedigree Best Linear Unbiased Prediction
ACD: after‐cooking darkening
GBLUP: Genomic Best Linear Unbiased Prediction
GBS‐t: genotyping‐by‐sequencing transcriptomics
GS: genomic selection
GWAS: genome‐wide association studies
HBLUP: Hybrid Best Linear Unbiased Prediction
MAS: marker‐assisted selection.

## 1 INTRODUCTION

Cultivated potato (*Solanum tuberosum* L.) is the fourth most important food crop in the world after the major grain crops of wheat, rice, and maize. The world's major potato producing regions are in Asia and Europe, accounting for more than 80% of global production. The high genetic diversity in potato has allowed for the identification of genotypes suited to various uses and diverse growing conditions. Almost all potato varieties grown across the globe have been developed following conventional breeding methods, i.e. hybridization, clonal propagation, evaluation and selection. The process can take more than 12 years, and considerable land and labour resources for the release of a new variety. Although potato breeding has resulted in the development of highly successful potato cultivars, it has been noted that there have been no significant improvements in the yield of potato cultivars over the last century (Barrell, Meiyalaghan, Jacobs, & Conner, 2013; Jansky, 2009) in comparison to grain crops (Hill, 2008). As a further challenge to plant breeders, shrinking land resources due to population growth and climate change add another layer of complexity to meet the future productivity of new cultivars.

Accelerating the genetic gain in potato using molecular tools is of great interest to potato breeders and for future global food security (Caruana et al., 2019; Endelman et al., 2018; Gebhardt, 2013; Slater et al., 2018; Slater, Cogan, Forster, Hayes, & Daetwyler, 2016; Slater, Wilson, Cogan, Forster, & Hayes, 2014b; Sverrisdóttir et al., 2018). Since the sequencing of the potato reference genome in 2011 (Potato Genome Sequencing Consortium 2011), the application of molecular marker techniques in potato breeding has become more accessible. The majority of the applications of marker‐assisted selection (MAS) have been limited to resistance screening against biotic stresses. Markers are available for disease resistance mediated by major R‐genes, as they are dominant traits and the presence/absence of the marker is often directly indicative of presence/absence of resistance. Traits of great interest, such as tuber yield and quality, are polygenic and complex, and using MAS for individual genetic effects for these traits would not be practical for potato breeding programs (Slater et al., 2014a). While significant genetic gains have been achieved for complex traits in diploid crops from high throughput genotyping methods and advanced genetic and statistical analysis (Crossa et al., 2010; Daetwyler, Bansal, Bariana, Hayden, & Hayes, 2014; Heffner, Jannink, & Sorrells, 2011), genomic data application in autopolyploids is still developing (Bourke, Voorrips, Visser, & Maliepaard, 2018; de Bem Oliveira et al., 2019). Autopolyploid inheritance is complex due to the presence of genotypes with higher allele dosage, a higher number of genotypic classes, poor knowledge of meiotic behaviour and multivalent formation (de Bem Oliveira et al., 2019; Sharma et al., 2018; Slater et al., 2014b). Next generation sequencing technologies have allowed for genome wide marker discovery that is less expensive, and new approaches for sequence‐based genotyping have been developed to implement genomic breeding approaches such as genomic prediction (Meuwissen, Hayes, & Goddard, 2001). Early genomic prediction results have shown the promise for rapid genetic progress in animals from selecting better individuals. Genomic prediction of breeding values has the potential to improve selection, reduce costs and provide a platform that unifies breeding approaches, biological discovery, and methods in both plant and animals breeding (Hickey, Chiurugwi, Mackay, & Powell, 2017). Nevertheless, effective utilization of genomic selection or prediction can be expensive and requires specialist skills (Hickey et al., 2017). Genomic predictions in different crop plants, including autopolyploids, using whole genome regression of phenotypes on sets of dense molecular markers is an active research area.

**Core Ideas**

- HBLUP can better leverage historic data for genomic selection or prediction than using only pedigree or genomics alone.
- HBLUP uses all data and ranks individuals on the same scale to ensure better selection decisions.
- Harnessing historical phenotypes through pedigrees was beneficial for genotyped individuals.

In crop breeding programs, the parental material and the breeding populations derived from them are generally large, consequently the genotyping costs are high. A breeding program would benefit if only a subset of individuals could be genotyped from a larger population without sacrificing genomic prediction accuracy as compared to using genetic information from the whole population. Use of genotypic information in place of pedigree information was first proposed in animals by Nejati‐Javaremi, Smith, and Gibson (1997) to predict breeding values. For the past decade, more importance was given to genotypic information to predict the breeding values of individuals, which provided much better estimates than using only pedigree information. However, in order to merge the information from both pedigree and genotype data, where a few individuals have genotypes and the rest only have pedigree information available, a unified single step computational approach for Genomic Best Linear Unbiased Prediction (ssGBLUP) for combining phenotypic, pedigree and genomic information was proposed and demonstrated in animals (Christensen & Lund, 2010; Legarra, Aguilar, & Misztal, 2009; Misztal, Legarra, & Aguilar, 2009) based on Henderson's (1975, 1976) standard mixed model equations. This method has been called HBLUP in crop breeding.

In HBLUP, the pedigree‐based relationship matrix of individuals is augmented with the genomic relationship matrix of genotyped individuals to obtain a hybrid (**H**) matrix. The **H** matrix therefore increases the connectedness in the genomic prediction reference population, especially for genotyped individuals and their relatives with incomplete pedigrees. This methodology could be of great interest in crops because it enables the inclusion of phenotype data from ungenotyped individuals in genomic prediction. This could be advantageous to make use of historical datasets where DNA is not available or when it is cost‐prohibitive to genotype all individuals of larger populations. To date two HBLUP studies have been published in crops (Ashraf et al., 2016; Pérez‐Rodríguez et al., 2017) and this is the first report of the method in an autotetraploid crop like potato.

In the present study we a) investigate the prediction accuracy of breeding values using either pedigree (ABLUP) or genomic information (GBLUP) on various potato traits; b) augment the numerator relationship matrix information from the pedigree data of a large set of individuals with genotypic data of a subset of genotypes (**H** matrix) to perform HBLUP, and c) compare the prediction accuracy of ABLUP, GBLUP and HBLUP in potato for different traits of economic interest.

## 2 MATERIALS AND METHODS

### 2.1 Populations and phenotypic data

Two phenotypic datasets were used for the present study. The first phenotypic data set contained three sets of breeding population progenies across two field generations (G2 and G3) evaluated from 2008 to 2011 and have been used earlier for estimating breeding values based on a pedigree matrix (Slater et al., 2014b). The second dataset was composed of 168 cultivars evaluated over several years prior to 2012 and used earlier for genomic predictions using various models (Caruana et al., 2019). In the breeding populations, the number of individuals varied across different traits due to higher tuber numbers in later generations allowing for more traits to be assessed.

The potato breeding populations were developed through hybridization under controlled conditions followed by germination and selection in the next generations. The derived true potato seeds were raised in trays and germinated seedlings transplanted to pots to raise the glasshouse (G0) generation. The plants were grown to maturity and a single tuber from each seedling pot was selected for field testing. The tubers were space planted in the first field generation (G1), each representing a unique individual. The G1 generation was manually harvested to maintain purity, and based on visual selection, the best plants were advanced to the next generation. All tubers were kept to enable up to 30 plants in the second field generation (G2). The G2 generation individuals were assessed for maturity and early blight resistance. The tubers from G2 generation plots were assessed for tuber shape and tuber eye depth at harvest, while selected individuals were assessed for cooking quality traits after harvest. Each individual was measured once for the traits. Selected individuals were then assessed in a third field generation (G3) replicated, randomised complete block trial for yield. Each plot consisted of 2 rows and was 5 m long with plant spacing varying depending on tuber size in the previous generation to mirror commercial practice. Each individual/parent only had a mean yield over replicates for further analyses.

The ‘07’ series contained 1,132 individuals across 57 families in the G2 trial in 2008, and 141 individuals in the G3 trial in 2009. The ‘08’ series had 1,137 individuals across 37 families in the G2 trial in 2009, and 138 individuals in the G3 trial in 2010. Similarly, the ‘09’ series had 952 individuals across 61 families in the G2 trial in 2010, and 112 individuals in the G3 trial in 2011. In brief, G2 trials were for phenotyping all the traits except yield, while G3 trials were for yield phenotyping solely. The series name represents the first year the series was planted in the field. Parents were used across multiple years, and each field trial had common check cultivars for performance comparison across years. Further details are described in Slater et al. (2014b).

The second dataset of cultivars contained data for more than 60 traits recorded in the Australian potato breeding program. The cultivars data were the result of the average of 3 years of trialling. The common traits from both of the datasets were selected for this study. The field trials for both the breeding populations and cultivars were conducted at the same field location in Toolangi, Victoria, Australia (37°34′ S, 145°30′ E, elevation ∼560 m).

The two phenotypic datasets were collected for different purposes and contained variations in the use of descriptive data, numerical data, or scale. For each trait the data was carefully examined and re‐scaled so that it could be compared and combined. To illustrate this, plant maturity was measured by visual observations on plants towards the end of life cycle in comparison to standard cultivars. The breeding population data set had 18 categories of maturity ranging from 0 for extremely late to 17 for very, very early, while the cultivars dataset observed nine categories from 1 for very, very early to 9 for very, very late. In order to compare the data, the scale of the 18 categories in the breeding population dataset was reversed and reduced to eight categories to align with the cultivar dataset (Supplemental Table S1a). The total number of individuals phenotyped for plant maturity was 3,545.

For the following traits any re‐scaling of data is presented in the supplementary file (Supplemental Table S1b‐d). Early blight resistance was visually scored towards the end of crop life cycle under natural infection conditions. The numerical score was from 1 for severe symptoms to 9 for absence of symptoms. The total number of individuals phenotyped for early blight resistance was 3,454. Tuber shape was visually scored at harvest on a scale of 1 for round to 8 for long taper/spindle/cigar. Tuber eye depth was visually scored on a scale of 1 for shallow to 6 for very deep. Tuber shape was scored for 1,636 individuals, while tuber eye depth was recorded for 1,637. Specific gravity was assessed through a comparison of the tubers weight in air compared with their weight in water. Average crisp score was assessed by cooking slices of tuber in oil at 180 °C for 2 min and they were then scored on a numerical scale from 1 for very light to 10 for very dark. For flesh colour, boil sloughing and after‐cooking darkening (ACD), tubers were peeled and boiled until cooked then boil sloughing scored from 1 for nil breakdown, to 5 for total breakdown, flesh colour was scored from 1 for white to 6 for deep yellow, and ACD was scored after 24 h from 1 for nil to 5 for very dark. The number of phenotyped individuals varied from 1,724–1,729 for these traits.

The tuber yield data in the breeding population dataset was assessed in the G3 generation in replicated trials in comparison to standard cultivars. Total tuber yield (tonnes/ha) was determined as fresh weight. The tuber yield data for the cultivars were scored visually. The individuals were placed in one of seven categories for total tuber yield. The tuber yield data of cultivars could not be merged with the data on the breeding populations, so only breeding population progenies yield data of 399 individuals were used in the analysis.

### 2.2 Pedigree information

Deep pedigree information was compiled from various sources for all the individuals in the merged dataset. The major source for pedigree information were our own pedigree database, the Potato Pedigree Database (van Berloo, Hutten, van Eck, & Visser, 2007) and European Cultivated Potato Database (https://www.europotato.org/). The information on US varieties was collected from individual varietal publications in American Journal of Potato Research. There were 261 founders, or individuals with no parental information, out of 4,179 total individuals in the pedigree. The pedigree file is available as supplementary Table S2.

### 2.3 Genotypic data

The Illumina sequence data of 168 potato cultivars was generated via a genotyping‐by‐sequencing transcriptomics GBS‐t protocol (Malmberg et al., 2017). The sequence data was aligned, subjected to filters, and a vcf file was generated using GATK tools (Caruana et al., 2019). Genotypes were extracted using the ‘vcfR’ package (version 1.8, Knaus and Grünwald (2018)) in R (Version 3.4.4, The R Foundation) from the same vcf file as Caruana et al. (2019). Tetraploid allele dosage calling was performed by counting the number of copies of a reference allele, where 0 denotes fully homozygous allele (AAAA), 1–3 represent heterozygous genotypes AAAB, AABB, ABBB, respectively, and 4 the other homozygous genotype (BBBB). The genotype file was processed in R to remove SNPs with more than two alleles, SNPs with missing data for more than 50 cultivars and with a minor allele frequency of < 0.03. The final file with genotypic information on 167 cultivars and 180,550 SNPs was used to generate the genomic relationship matrix (**G**).

### 2.4 A, G and H matrices and the corresponding BLUP

The pedigree‐based relationship matrix (**A**) was built considering an autotetraploid model with 10 per cent double reduction (Slater et al., 2014b). The details of the approach for the numerator relationship matrix can be seen in Slater et al. (2014b). **G** was generated using method 1 in Van Raden (2008). The R package ‘AGHmatrix’ (Amadeu et al., 2016) was used to obtain these two matrices. The hybrid matrix (**H**) that combined information from **A** and **G** (Legarra et al., 2009) was derived using the method in Pérez‐Rodríguez et al. (2017). Heatmaps of the relationship were plotted using ‘superheat’ R package. Relationships between genotyped cultivars were investigated from **G** as well as the subset of corresponding lines from **A** and **H**. Solely for plotting heatmaps, the **G** and **H** matrices were adjusted to be on the same scale by subtracting the minimum values in the matrix before plotting, so that the minimum value was 0 for all three matrices to enable comparisons.

Pedigree based best linear unbiased prediction (ABLUP) is a model predicting breeding values using the expected relatedness among individuals (**A**) (Henderson, 1984), while genomic best linear unbiased prediction (GBLUP) is used to predict genomic breeding values by replacing **A** with **G**. In addition, HBLUP is a model applying a hybrid matrix (**H**) to predict breeding values based on pedigree, phenotypes and genotypes.

### 2.5 Mixed linear model

Single‐trait linear mixed models were used to predict breeding values using the restricted maximum likelihood approach (REML) as follows:

$$y=1_{n}\mu +\mathrm{Xb}+\mathrm{Zu}+e$$

where, **y** is a vector of phenotypic records for the particular trait, μ is the overall mean, **1_n_** is a vector of ones, **b** is a vector of fixed effects (i.e. year), **X** is a matrix allocating records to the effects, **Z** is a matrix allocating records to breeding values, **u** is a vector of breeding values, distributed as N(0,**M**σ^2^_g_), where **M** denotes **A**, **G** or **H** in ABLUP, GBLUP and HBLUP, respectively, and σ^2^_g_ is the respective genetic variance captured by the associated matrix, **e** is a vector of random error terms distributed as N(0, **I**σ^2^_e_), **I** denotes an identity matrix and σ^2^_e_ is the error variance. The R package Sommer was used to fit all predictions (Covarrubias‐Pazaran, 2016).

### 2.6 Cross validation schemes

**FIGURE 1:**
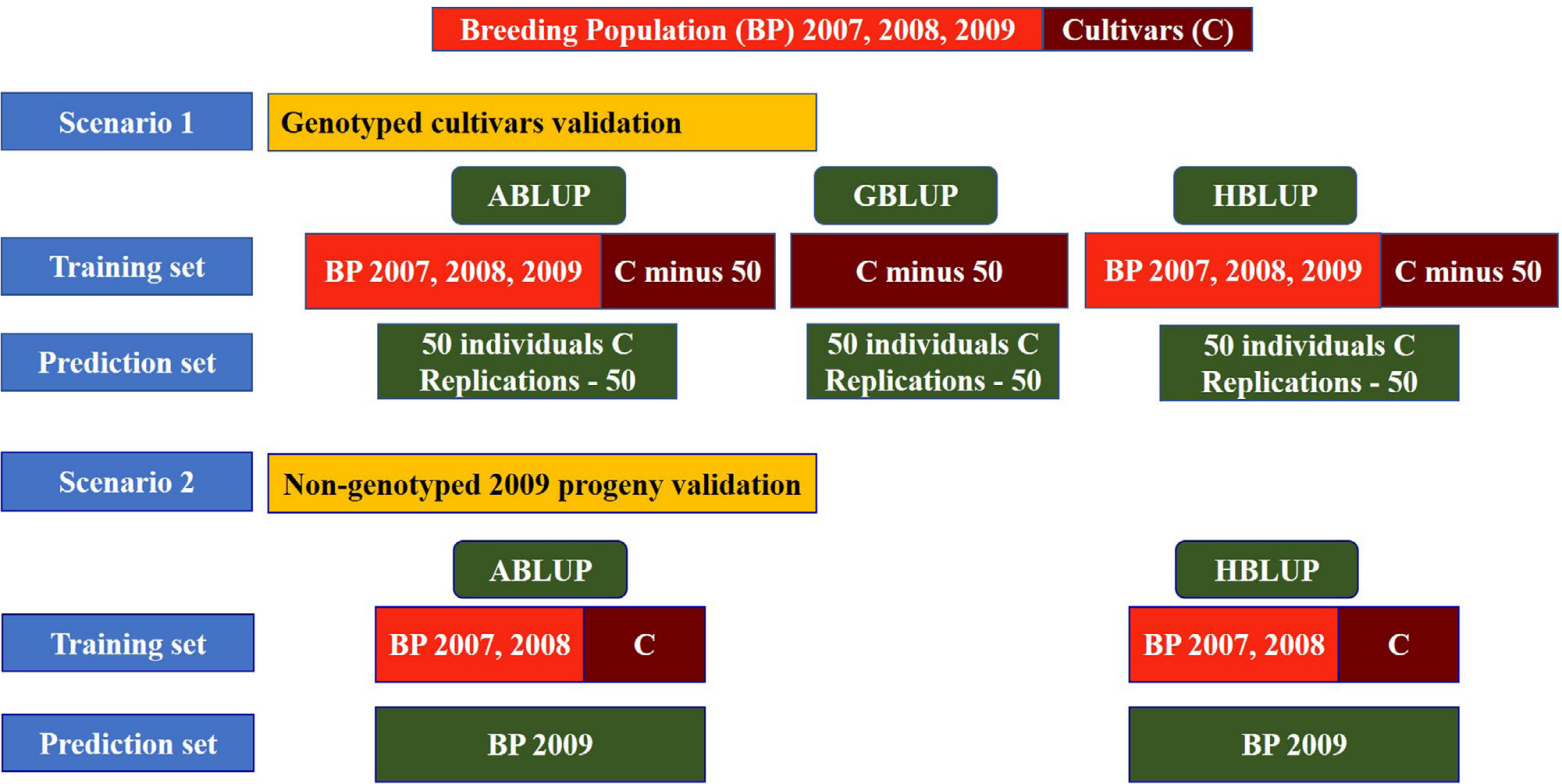
Description of the two main genomic prediction scenarios using best linear unbiased prediction (BLUP) with pedigree (A), genomic (G) and hybrid (H) relationship matrices

Two scenarios of cross validation were applied to test the prediction accuracies from different BLUP models. The prediction accuracies were estimated as the Pearson correlation coefficient between the genetic estimated breeding values and the observed phenotypic values per trait. The first scenario (genotyped cultivars validation) was to randomly sample 50 genotyped cultivars as a validation set, while using the remaining genotyped cultivars as a training set in GBLUP, and the remaining genotyped cultivars plus progenies as the training set in ABLUP/HBLUP and 50 replicates. The fixed effect in ABLUP/HBLUP was the years of the G2 trials (2008, 2009 and 2010) of the three series (‘07’, ‘08’ and ‘09’). The second scenario (Non‐genotyped 2009 progeny validation) assigned all of the progenies from 2009 into a validation set and used the remaining progenies as well as genotyped cultivars as training set in both ABLUP and HBLUP (Figure 1). Year information of the G3/cultivar trials except for the ‘09’ series (the validation set) was fitted as a fixed effect. Bias was investigated as the slope of the regression of phenotypes on (G)EBV for both scenarios, where the results were the mean (s.e.) across 50 replicates per trait for the genotyped cultivars validation, while one regression coefficient per trait for the Non‐genotyped 2009 progeny validation. In addition, narrow‐sense heritabilities were estimated for all the traits via HBLUP by fitting all available phenotypes in the model.

### 2.7 Data availability

Phenotypic data used in the analyses are available in Supplemental Table S3, while genotypic data are available in Caruana et al. (2019). All data necessary for confirming the conclusions of the article are publicly available or present within the article, figures, and tables.

## 3 RESULTS

### 3.1 Phenotype and genotypes

There was a wide phenotypic range for all the traits in the combined dataset (breeding populations and cultivars; Supplemental Table S4; Supplemental Figure S1). Individual genotypes spanned the five categories of boil ACD, although the vast majority had lower ACD scores, as this is the desirable trait (Supplemental Figure S1e). For average crisp score, the trait distribution varied from 2–10, where a lower score indicates a lighter and better crisp, while a higher value indicates a darker undesirable crisp (Supplemental Figure S1g). Similarly, for boil sloughing, there was representation of individuals in all the categories ranging from 1 of no sloughing to 5 a total breakdown (Supplemental Figure S1f). The number of individuals in each category for maturity also followed a normal distribution (Supplemental Figure S1j). Early blight score varied from 1 for severe symptoms to 9 for no symptoms, with most individuals in the middle followed by individuals towards the lower score and least in the higher score (Supplemental Figure S1i). Flesh colour score varied from 1 for white to 5 for deep yellow and exhibited a similar pattern to that of early blight (Supplemental Figure S1d). Tuber eye depth and tuber shape score showed right skewed distribution indicating most individuals had shallow eye depth and round oval to oval tubers (Supplemental Figure S1b,c). Specific gravity values followed a normal distribution and the range of variation was 1.053–1.119 (Supplemental Figure S1h).

### 3.2 Relationship matrices

**FIGURE 2:**
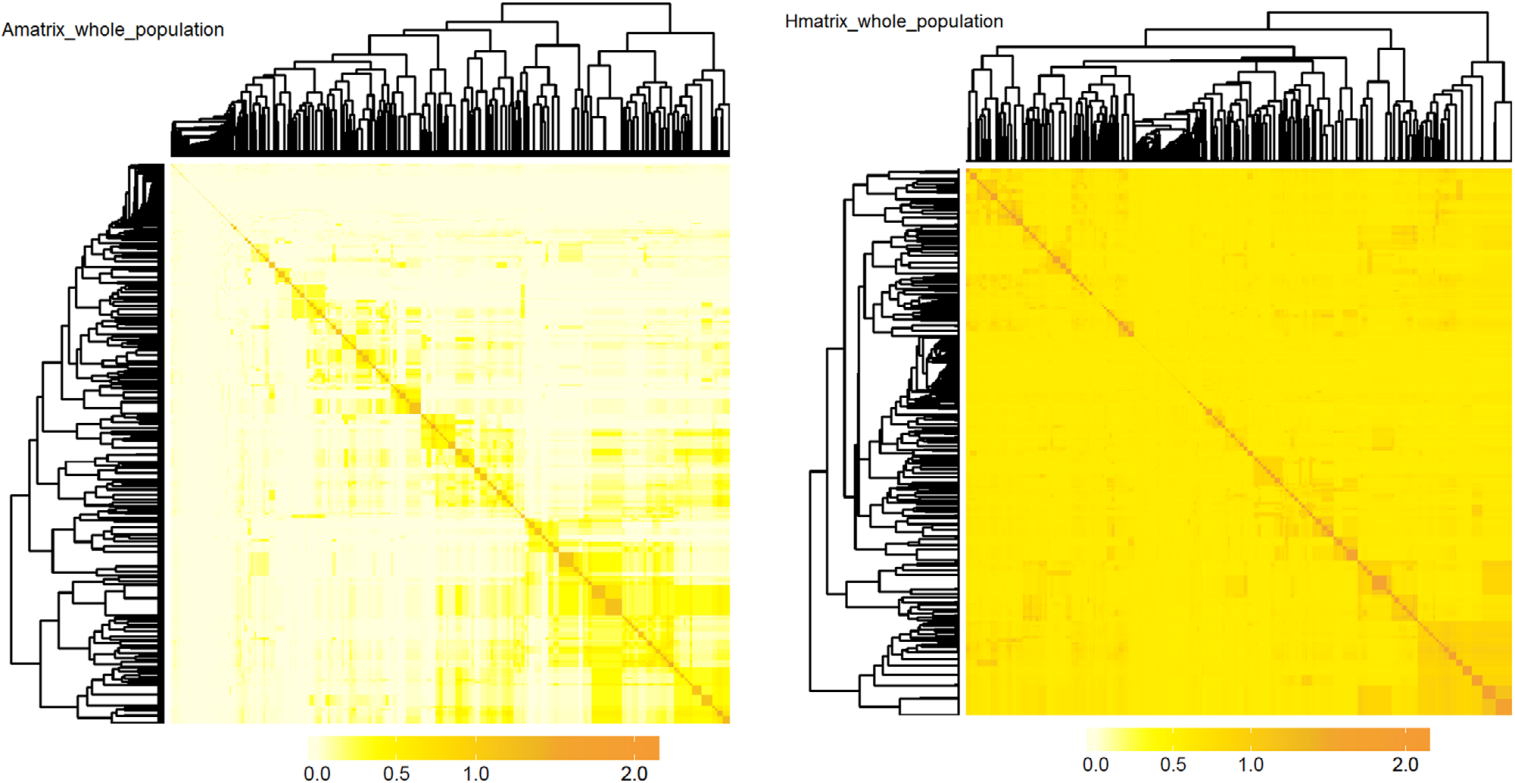
Heatmaps of **A** (left panel) and **H** (right panel) for genotyped cultivars and progenies, where darker colour indicates higher relatedness

The relationship among progenies and genotyped cultivars was investigated via heatmaps of **A** and **H** (Figure 2), where compared to **A**, a stronger relationship was found in **H** with the additional information from the genomic data of cultivars. The relationship among genotyped cultivars was investigated via heatmaps of **A**, **G** and **H** (Supplemental Figure S2). As shown in the histogram of Supplemental Figure S2a, the majority of the relationship captured by **A** was between 0 and 0.1. The relationship among the same populations was found to be stronger in **G** with approximately 20,000 pairwise relationship between 0.1 and 0.3, and further increased in **H** with 24,000 pairwise relationship between 0.1 and 0.3. In addition, scatter plot of the off‐diagonal elements was created for the corresponding lines between **G** and **A** (Supplemental Figure S3).

### 3.3 Heritabilities

The heritabilities estimated for different traits varied from 0.22 (Plant maturity) to 0.72 (tuber shape) using **H**. Tuber shape (0.72), flesh colour (0.59), specific gravity (0.55) and average crisp score (0.52) recorded high heritabilities, while other traits showed moderate to low heritabilities (Table 1).

**TABLE 1:** Narrow sense heritabilities estimated using HBLUP (**H** matrix)

Traits	Heritability
Total tuber yield	0.37
Tuber shape	0.72
Tuber eye depth	0.42
Flesh color	0.59
Boil ACD	0.35
Boil sloughing	0.29
Average crisp score	0.52
Specific gravity	0.55
Early blight	0.47
Plant maturity	0.22

### 3.4 Genomic prediction

#### 3.4.1 Genotyped cultivars validation

(G)EBVs were estimated for the 50 randomly sampled cultivars per replicate using ABLUP, GBLUP, and HBLUP, respectively. Prediction accuracies (Table 2) varied from 0.10 for plant maturity to 0.68 for flesh colour in ABLUP. Besides flesh colour (0.68), specific gravity (0.44), tuber eye depth (0.40), boil sloughing (0.36) and tuber shape (0.32) also observed moderate to high accuracies, while low prediction accuracies were achieved for early blight (0.20), boil ACD (0.18), average crisp score (0.15) and plant maturity (0.10). As compared to ABLUP, accuracies were significantly increased in GBLUP for average crisp score (0.15 vs. 0.34), plant maturity (0.10 vs. 0.25), flesh colour (0.68 vs. 0.77), specific gravity (0.44 vs. 0.52), boil sloughing (0.36 vs. 0.41) and tuber eye depth (0.40 vs. 0.43) (Table 2). The highest accuracy was again observed for flesh colour (0.77), while the lowest accuracy was found for early blight (0.14). Prediction via HBLUP resulted in higher accuracy in comparison to ABLUP for all the traits except boil sloughing (both 0.36) and early blight (0.10 vs. 0.20). Moreover, HBLUP was superior over GBLUP on prediction accuracies for tuber shape (0.37 vs. 0.28), tuber eye depth (0.49 vs. 0.43) and boil ACD (0.27 vs. 0.20), while it achieved comparable or lower accuracies for the other traits (Table 2).

**TABLE 2:** Training population sizes and the prediction accuracies for traits in the genotyped cultivars validation. Results are means ± s.e. across 50 replicates

	Training population size		Prediction accuracy		
Traits	GBLUP	ABLUP and HBLUP	ABLUP	GBLUP	HBLUP
Tuber shape	103	1591	0.32 ± 0.019	0.28 ± 0.016	0.37 ± 0.015
Tuber eye depth	104	1595	0.40 ± 0.017	0.43 ± 0.012	0.49 ± 0.012
Flesh color	88	1676	0.68 ± 0.010	0.77 ± 0.007	0.75 ± 0.008
Boil ACD	90	1678	0.18 ± 0.013	0.20 ± 0.015	0.27 ± 0.014
Boil sloughing	90	1678	0.36 ± 0.016	0.41 ± 0.016	0.36 ± 0.013
Average crisp score	88	1680	0.15 ± 0.016	0.34 ± 0.012	0.34 ± 0.011
Specific gravity	91	1681	0.44 ± 0.009	0.52 ± 0.009	0.53 ± 0.007
Early blight	65	3405	0.20 ± 0.016	0.14 ± 0.013	0.10 ± 0.019
Plant maturity	79	3495	0.10 ± 0.010	0.25 ± 0.014	0.21 ± 0.008
Mean	‐	‐	**0.31 ± 0.01**	**0.37 ± 0.01**	**0.38 ± 0.01**

#### 3.4.2 Non‐genotyped 2009 progenies validation

The predictions accuracies of (G)EBVs were investigated on the non‐genotyped 2009 progenies via ABLUP and HBLUP (Table 3). In general, the prediction accuracies achieved on 2009 progenies were comparable between ABLUP and HBLUP for all the traits without a considerable increase or decrease. Prediction accuracies were further investigated on the non‐genotyped 2009 individuals which were progeny or grand‐progeny of genotyped parents and were found to be marginally increased compared to the accuracies for the whole population across all the traits (Table 3).

**TABLE 3:** Training population sizes and prediction accuracies on all/subset of non‐genotyped 2009 progenies

		Accuracy on all non‐genotyped 2009 progenies		Accuracy on non‐genotyped 2009 progenies with close relatedness to genotyped cultivars	
Trait	Training population size	ABLUP	HBLUP	ABLUP	HBLUP
Total tuber yield^A^	413	0.30	0.32	0.33	0.34
Tuber shape^B^	1140	0.69	0.68	0.70	0.69
Tuber eye depth^B^	1141	0.47	0.50	0.50	0.53
Flesh color^B^	1232	0.61	0.60	0.64	0.63
Boil ACD^B^	1234	0.27	0.29	0.27	0.29
Average crisp score^B^	1235	0.48	0.51	0.47	0.50
Boil sloughing^B^	1235	0.57	0.56	0.56	0.56
Specific gravity^B^	1236	0.70	0.69	0.70	0.68
Early blight^C^	2510	0.18	0.18	0.20	0.21
Plant maturity^C^	2593	0.17	0.19	0.18	0.20
**Mean**	‐	**0.44**	**0.45**	**0.46**	**0.46**

*Note*. No. in validation sets: ^A^ 100, ^B^ 500, and ^C^ 950 for ‘all 2009 progenies’, and subtracting ∼50 for ‘the 2009 progenies which having relatedness with genotyped cultivars’.

The regression coefficient of phenotypes on (G)EBVs were investigated for both scenarios (Tables 4 and 5). As shown in Table 4 (genotyped cultivars validation), the regression coefficients from most of the traits are distant to 1, except tuber shape which was relatively close to 1 across the three BLUP models. The overall mean of regression coefficient from HBLUP was the one closest to 1 as compared to the ones from ABLUP and GBLUP. In addition, regression coefficients estimated in the scenario of non‐genotyped 2009 progeny validation (Table 5) were close to one for tuber eye depth, flesh colour, tuber shape and average crisp score. Regression coefficients for boil sloughing and boil ACD showed large deviations from one. However, the overall means of regression coefficients were comparable between ABLUP and HBLUP in this scenario (1.25 vs. 1.24, Table 5).

**TABLE 4:** Regression coefficients of phenotypes on (G)EBV for the random genotyped cultivars validation set, means ± s.e. across 50 replicates per trait

**Traits**	**Regression coefficient with EBV of ABLUP**	**Regression coefficient with GEBV of GBLUP**	**Regression coefficient with GEBV of HBLUP**
Tuber shape	1.07 ± 0.03	1.02 ± 0.02	1.18 ± 0.02
Tuber eye depth	1.32 ± 0.03	1.35 ± 0.04	1.57 ± 0.02
Flesh color	1.42 ± 0.00	1.29 ± 0.00	1.33 ± 0.00
Boil ACD	0.43 ± 0.01	1.67 ± 0.06	0.69 ± 0.02
Average crisp score	0.49 ± 0.02	1.75 ± 0.13	1.10 ± 0.01
Boil sloughing	0.64 ± 0.01	1.27 ± 0.06	0.65 ± 0.00
Specific gravity	1.04 ± 0.01	1.53 ± 0.02	1.19 ± 0.01
Early blight	0.84 ± 0.04	1.96 ± 0.13	0.45 ± 0.06
Plant maturity	0.65 ± 0.04	1.58 ± 0.12	1.38 ± 0.03
**Overall mean** (s.e.)	**0.88 ± 0.12**	**1.49 ± 0.10**	**1.05 ± 0.12**

**TABLE 5:** Regression coefficients of phenotypes on (G)EBV for the non‐genotyped 2009 progenies validation set

**Traits**	**Regression coefficient with EBV of ABLUP**	**Regression coefficient with GEBV of HBLUP**
Total tuber yield	1.29	1.41
Tuber shape	1.18	0.93
Tuber eye depth	0.98	0.98
Flesh color	0.85	0.84
Boil ACD	1.73	1.78
Average crisp score	1.23	1.19
Boil sloughing	3.02	2.89
Specific gravity	1.30	1.31
Early blight	0.54	0.59
Plant maturity	0.33	0.43
**Overall mean**	**1.25**	**1.24**

## 4 DISCUSSION

This study demonstrates the application of genomic selection in autotetraploid potato by combining genotyped and non‐genotyped individuals from two previously published studies to investigate the potential prediction accuracy (Caruana et al., 2019; Slater et al., 2014b). In general, the prediction accuracies were moderate to high for most of the traits with high heritabilities and vice versa, except early blight in both the scenarios. HBLUP outperformed GBLUP for three traits in genotyped cultivars prediction, while the accuracies of ABLUP and HBLUP were at par for most traits in non‐genotyped 2009 progeny prediction. The inclusion of genetic marker information in the **G** and **H** matrices increased the relatedness amongst the lines. This increased connectedness in the combined **H** matrix was expected to increase the analytical power and increased prediction accuracy in both validation scenarios. However, the increase in prediction accuracy was modest using HBLUP, but, overall, using historical data for prediction was observed to be beneficial for genomic prediction. In addition, computational times were comparable between all three BLUP models in this study.

We observed a wide range of phenotypic variation for all traits in the merged dataset, which is the prerequisite for any selection program in crop breeding, and populations with high genotypic variance tend to display better genomic selection accuracy (Crossa et al., 2013). High narrow sense heritability for a trait indicates that most of the phenotypic variance is under genetic control and selection will be effective. Flesh colour and tuber shape is expected to show high heritability as the trait is governed by few genes and is not strongly affected by environmental conditions (Slater et al., 2014a). Tuber eye depth should not be affected greatly by growing conditions and should express high heritability, however we observed moderate heritability. Specific gravity and average crisp score could be influenced by environmental growing conditions and soil nutritional status. Early blight observed moderate heritability as the early blight ratings are affected by variable inoculum levels in different years. Low heritabilities estimated for plant maturity, boil sloughing and boil ACD indicates higher environmental influence and non‐additive genetic components for these traits. The low heritability estimated for plant maturity could be due to seasonal variation and disease pressure. Our result of heritability for plant maturity was in contrast to Slater et al. (2014b) and Stich and Van Inghelandt (2018), who observed high estimates of heritability for plant maturity. The heritability estimated for flesh colour, specific gravity, average crisp score and early blight were similar to Slater et al. (2014b), and for average crisp score similar to Sverrisdóttir et al. (2017)). In accordance with our study, Ticona‐Benavente and da Silva Filho (2015) also observed low heritability for tuber yield and high heritability for specific gravity.

High prediction accuracies of genotyped cultivars for most traits except early blight and tuber shape in GBLUP over ABLUP are attributed to more variance captured by **G** than with pedigree information. The predictions based on genotype data should do better than pedigree based method because they can account for and predict the effects of Mendelian segregation (Ashraf et al., 2016). Endelman et al. (2018) also reported superiority of GBLUP over ABLUP for tuber yield and fry colour, which is the same as our trait average crisp score. The prediction accuracies for cultivars in HBLUP were higher or at par for most traits except boil sloughing, early blight, and plant maturity in comparison to GBLUP (Table 2). Most cultivars had pedigree information and their genomic relationship information could flow deep into the pedigree, resulting in increased accuracy from HBLUP. The improved relationship in the **G** subset of the **H** matrix in comparison to **A** matrix is evident in the heatmaps (Supplemental Figure S2). The increased relationship captured more additive variation and likely to result in enhanced prediction accuracy in HBLUP. Moreover, usage of **H** allowed non‐genotyped individuals to join model training for genomic prediction. The size of training population could therefore be increased (e.g. training size of ∼100 in GBLUP increased to >2000 in HBLUP in our study), leading to potential enhancements to prediction accuracies using HBLUP as compared to GBLUP.

In the second scenario (non‐Genotyped 2009 Progeny Validation), the prediction accuracy of ABLUP and HBLUP were similar (Table 3). In addition, prediction accuracies differed little between progenies regardless of whether they had genotyped parents or not. We would have expected an increase in the accuracy from HBLUP due to better capturing the relatedness between lines, as was evident from the heatmaps of the **A** and **H** relationship matrices (Figure 2), however, the improvement was minimal. One reason may be that most progenies had parents with phenotypes and that adding genotypes through **H** may be more beneficial for lines whose parents do not have phenotypes. Another reason could be that the additional information provided by **G** was small due to a large difference of population size between **G** and **A** (167 and 4179 individuals, respectively). Similarly, non‐significant differences were observed among HBLUP and ABLUP for prediction accuracy of wheat grain yield across locations (Pérez‐Rodríguez et al., 2017). Another study found HBLUP outperformed ABLUP for all the traits in fivefold cross validations. However, standard errors on accuracies were not reported so it is difficult to judge whether the differences were significant (Ashraf et al., 2016). In addition, our study only tested a single weight on **G** to obtain **H**. Further work could investigate various weights or even trait‐specific weights to obtain a more optimal **H**.

The level of prediction accuracy for different traits in both the scenarios were similar to previous studies (Caruana et al., 2019; Enciso‐Rodriguez, Douches, Lopez‐Cruz, Coombs, & de Los Campos, 2018; Endelman et al., 2018; Slater et al., 2016; Stich & Van Inghelandt, 2018; Sverrisdóttir et al., 2017; Sverrisdóttir et al., 2018) on genomic selection in potato, except plant maturity and disease resistance. Stich and Van Inghelandt (2018) observed moderate (0.5) to high (0.8) cross prediction accuracies for six key performance traits including tuber yield using GBLUP and three Bayesian approaches, and suggested the use of Bayesian methods for oligogenic traits. Enciso‐Rodriguez et al. (2018) observed prediction correlation range of 0.41–0.74 using large populations. We observed low prediction accuracy for plant maturity, while high prediction accuracies were observed previously (Slater et al., 2016; Stich & Van Inghelandt, 2018). Low heritability of late maturity due to merger of two different datasets could be the probable reason for low prediction accuracy. This could be substantiated with two earlier studies, breeding population dataset (Slater et al., 2016) and cultivars dataset (Caruana et al., 2019), which observed high and low prediction accuracy for plant maturity, respectively. Similarly, low genomic prediction accruacy was also found for early blight. Although disease resistance is expected to show high prediction accuracy as observed for late blight (Enciso‐Rodriguez et al., 2018; Stich & Van Inghelandt, 2018), low prediction accuracy for early blight resistance despite moderate heritability estimate is consonant with the observation that for early blight the relationship between expected and observed values was especially low due to inconsistency in the phenotypic score of the disease resistance measured for the two populations (progenies and cultivars). Finally, moderate accuracies were observed for specific gravity and fry colour, which were comparable to other studies (Endelman et al., 2018; Sverrisdóttir et al., 2018).

For the successful application of genomic selection/prediction must be accurate and unbiased. A coefficient near one indicates that the (G)EBVs are neither over or underestimated when compared against phenotypes. This is important when combining (G)EBVs from different datasets for ranking purposes. Compared to ABLUP and GBLUP, our study found HBLUP had the least bias on prediction in the genotyped cultivars validation. While in the non‐genotyped 2009 progeny validation, the averaged bias across traits was approximately 1.25 using HBLUP, which is in the acceptable range.

Overall, our study found that the prediction accuracies were in the usable range and suggest that substantial genetic gain could be achieved for potato traits per unit of time and cost using the GBLUP model (Slater et al., 2016). The level of accuracy achieved indicate that parental cultivars should be part of the reference population to ensure sufficient prediction accuracy and this would allow for use of GEBVs in glasshouse seedlings. The accuracy in the second and third generation could be further improved by sparse phenotyping a subset of progenies for inclusion in the reference population. Computer simulations could be used to shed more light on how to increase genetic gain in genomic potato breeding programs, which has been demonstrated in other plant species (Iwata & Jannink, 2011; Lin et al., 2016; Yabe, Ohsawa, & Iwata, 2013).

There are several benefits from implementing HBLUP for breeding. First, it can increase the size of training population for genomic prediction by incorporating phenotypes from non‐genotyped individuals. This is especially attractive for a breeding program (i.e. potato breeding) to get the most from its non‐genotyped historical germplasm, while only a small number of samples are genotyped. HBLUP enables the use of historical data on non‐genotyped individuals and could also be used to combine information from different breeding programs and research groups. Second, cost savings could be realised by only genotyping the parents and a subset of the progenies rather than genotyping the entire breeding population, and then using HBLUP to predict the better families and individuals for further evaluation. Third, HBLUP ensures that all breeding values are on the same scale and can be ranked easily to facilitate breeding decisions, without having to blend pedigree and genomic breeding values that are potentially estimated on different scales. Four, the method is practical, as only one evaluation is needed for both genotyped and non‐genotyped individuals making use of all available information to increase power and potentially reduce bias. However, the inconsistency of phenotyping across datasets may present one of the largest hurdles when combining different data sources. Further research is needed to more optimally standardise phenotype datasets. In terms of genomic data, the combination of datasets genotyped using different platforms also presents challenges, not only in finding sufficient overlapping markers but also due to differences in scoring autotetraploid genotypes. Aside from using exactly the same genotyping platform, whole‐genome sequencing and imputation may be able to link genotype datasets going forward, but imputation of autopolyploids also requires method development. Finally, in very large datasets and when non‐genotyped individuals outnumber genotyped ones, the inversion of **H** becomes computationally challenging. However, methods are developed for animal populations that are already reaching those limits (Boerner & Johnston, 2019; Misztal, 2016).

## AUTHOR CONTRIBUTIONS

SS and ZL conducted the analysis. BC analysed and provided the sequencing data. AS provided the phenotypic data and local pedigree data. SS, ZL, HD, AS conceived and designed the study, and all authors contributed to writing and approved the final manuscript.

## CONFLICT OF INTEREST

The authors declare that they have no conflict of interest.

## Supporting information

Table S1: Realignment of dataset scales

Table S2: Pedigree file of individuals used for creating pedigree relationship matrix

Table S3: Trait wise phenotypic data used in the analysis

Table S4: Summary statistics of cultivars and breeding populations dataset

Fig. S1a. Frequency distribution of individuals for total tuber yield in breeding population dataset

Fig. S1b. Frequency distribution of individuals for tuber shape in combined dataset

Fig. S1c. Frequency distribution of individuals for tuber eye depth in combined dataset

Fig. S1d. Frequency distribution of individuals for tuber flesh colour in combined dataset

Fig. S1e. Frequency distribution of individuals for boil ACD in combined dataset

Fig. S1f. Frequency distribution of individuals for boil sloughing in combined dataset

Fig. S1g. Frequency distribution of individuals for average crisp score in combined dataset

Fig. S1h. Frequency distribution of individuals for specific gravity in combined dataset

Fig. S1i. Frequency distribution of individuals for early blight in combined dataset

Fig. S1j. Frequency distribution of individuals for plant maturity in combined dataset

Fig. S2a. Heatmap of A for the genotyped cultivars

Fig. S2b. Heatmap of G for the genotyped cultivars

Fig. S2c. Heatmap of H for the genotyped cultivars

Fig. S3. Scatter plot of the off‐diagonal elements for the corresponding lines between G and A
