# Supplementary material for: Making the most of all data: Combining non‐genotyped and genotyped potato individuals with HBLUP
Source: Plant Genome. 2020 Sep 29;13(3):e20056. doi: 10.1002/tpg2.20056 (PMC12807232; doi:10.1002/tpg2.20056)
Supplement: Supplementary file 1 [file TPG2-13-e20056-s001.docx]

**Table S4: Summary statistics of cultivars and breeding populations dataset**

| **Traits** | **Number of records*** | | **Mean** | | **Min** | | **Max** | | **SD** | |
| --- | --- | --- | --- | --- | --- | --- | --- | --- | --- | --- |
|  | Cultivars | Breeding populations | Cultivars | Breeding populations | Cultivars | Breeding populations | Cultivars | Breeding populations | Cultivars | Breeding populations |
| Total Tuber Yield | - | 424 | - | 53.34 | - | 22.04 | - | 82.8 | - | 10.14 |
| Tuber Shape | 169 | 1472 | 3.56 | 3.20 | 1 | 1 | 8 | 7 | 1.99 | 1.58 |
| Tuber Eye Depth | 170 | 1472 | 1.95 | 1.68 | 1 | 1 | 5 | 6 | 1.22 | 1.03 |
| Flesh Colour | 153 | 1573 | 2.29 | 2.64 | 1 | 1 | 5.5 | 5.5 | 1.51 | 1.23 |
| Boil ACD | 156 | 1572 | 1.61 | 1.79 | 1 | 1 | 5 | 5 | 0.58 | 0.87 |
| Boil Sloughing | 156 | 1572 | 1.96 | 2.39 | 1 | 1 | 4 | 5 | 0.61 | 1.12 |
| Average Crisp Score | 154 | 1576 | 5.93 | 7.06 | 3 | 2 | 10 | 10 | 1.40 | 1.54 |
| Specific Gravity | 157 | 1574 | 1.08 | 1.09 | 1.053 | 1.054 | 1.106 | 1.119 | 0.009 | 0.012 |
| Early blight | 128 | 3327 | 3.24 | 4.48 | 1 | 1 | 8 | 9 | 2.21 | 2.17 |
| Plant Maturity | 143 | 3402 | 5.13 | 5.42 | 2 | 2 | 9 | 8 | 1.52 | 1.17 |

*-includes repeated entries also
