# Supplementary material for: Making the most of all data: Combining non‐genotyped and genotyped potato individuals with HBLUP
Source: Plant Genome. 2020 Sep 29;13(3):e20056. doi: 10.1002/tpg2.20056 (PMC12807232; doi:10.1002/tpg2.20056)
Supplement: Supplementary file 2 [file TPG2-13-e20056-s018.docx]

**Table S1: Realignment of dataset scales**

a) **Realignment of the dataset scales for comparison of the maturity scores.**

| **Scale description** | **Breeding populations dataset scale** | **Cultivars dataset scale** | **Final numerical scale** |
| --- | --- | --- | --- |
| Very, very late | - | 9 | 9 |
| Very late | 0 (very late^+^) | 8 | 8 |
|  | 1 (very late) |  |  |
|  | 2 (very late^-^) |  |  |
| Late | 3 (late^+^) | 7 | 7 |
|  | 4 (late) |  |  |
|  | 5 (late^-^) |  |  |
| Medium late | 6 (medium late^+^) | 6 | 6 |
|  | 7 (medium late) |  |  |
|  | 8 (medium late^-^) |  |  |
| Medium | 9 (medium^+^) | 5 | 5 |
|  | 10 (medium) |  |  |
|  | 11 (medium^-^) |  |  |
| Medium early | 12 (medium early^+^) | 4 | 4 |
|  | 13 (medium early) |  |  |
|  | 14 (medium early^-^) |  |  |
| Early | 15 (early) | 3 | 3 |
| Very early | 16 (very early) | 2 | 2 |
| Very, very early | 17 | 1 | 1 |

b) **Realignment of the dataset scales for comparison of the early blight resistance scores**

| **Scale description** | **Breeding population scale** | **Cultivars scale** | **Changed cultivars scale** | **Final scale** |
| --- | --- | --- | --- | --- |
| Severe | 1 | 6 | 1 | 1 |
| Medium severe | 2 | 5 | 2 | 2 |
| Moderate^+^ | 3 | - | - | 3 |
| Moderate | 4 | 4 | 4 | 4 |
| Slightly moderate | 5 | 3 | 5 | 5 |
| Slight | 6 | 2 | 6 | 6 |
| Slight to negligible | 7 | - | - | 7 |
| Negligible | 8 | 1 | 8 | 8 |
| Nil | 9 | - | - | 9 |

c) **Realignment of the dataset scales for comparison of the flesh colour scores**

| **Scale description** | **Breeding population scale** | **Cultivars scale** | **Changed cultivars scale** | **Final scale** |
| --- | --- | --- | --- | --- |
| White | 1 | 1 | 1 | 1 |
| White - cream | 2 | 2 | 2 | 2 |
| Cream | 3 | 3 | 3 | 3 |
| Cream-light yellow | 4 | 4 | 4 | 4 |
|  |  | 5 |  |  |
|  |  | 6 |  |  |
| Yellow | 5 | 7 | 5 | 5 |
| Dark yellow | 6 | 8 | 6 | 6 |

d) **Realignment of the dataset scales for comparison of the tuber shape scores**

| **Scale description** | **Breeding populations scale** | **Revised breeding population scale** | **Cultivars scale** | **Revised Cultivars datatset scale** | **Final scale** |
| --- | --- | --- | --- | --- | --- |
| Round | 1 | 1 | 1 | 1 | 1 |
| Round-oval | 2 | 2 | 2 | 2 | 2 |
| Oval | 3 | 3 | 3 | 3 | 3 |
| Oval-oblong | 4 | 4 | 4 | 6 | 4 |
| Oblong | 5 | 5 | 5 | 4 | 5 |
| Oval- long | 6 | 6 | 6 | 5 | 6 |
| Oblong-long | 7 | 6 | 7 | 6 |  |
| Long | 8 | 7 | 8 | 7 | 7 |
| Long taper/spindle/cigar | 9 | 8 | 9 | - | 8 |
